# Poly(N-Isopropylacrylamide) Based Electrically Conductive Hydrogels and Their Applications

**DOI:** 10.3390/gels8050280

**Published:** 2022-05-01

**Authors:** Zexing Deng, Yi Guo, Xin Zhao, Tianming Du, Junxiong Zhu, Youlong Xie, Fashuai Wu, Yuheng Wang, Ming Guan

**Affiliations:** 1 College of Materials Science and Engineering, Xi’an University of Science and Technology, Xi’an 710054, China; 2 Shaanxi Key Laboratory of Brain Disorders, Shaanxi Key Laboratory of Ischemic Cardiovascular Disease, Institute of Basic and Translational Medicine, Xi’an Medical University, Xi’an 710021, China; guoyi@xiyi.edu.cn; 3 State Key Laboratory for Mechanical Behavior of Materials, Frontier Institute of Science and Technology, Xi’an Jiaotong University, Xi’an 710049, China; zhaoxinbio@mail.xjtu.edu.cn; 4 Beijing International Science and Technology Cooperation Base for Intelligent Physiological Measurement and Clinical Transformation, Department of Biomedical Engineering, Faculty of Environment and Life, Beijing University of Technology, Beijing 100124, China; dutianming@bjut.edu.cn; 5 Department of Orthopedics Surgery, The Second Affiliated Hospital, Zhejiang University School of Medicine, Hangzhou 310058, China; junxiong23@163.com; 6 Department of Reproductive Biology, School of Public Health and Management, Chongqing Medical University, Chongqing 400042, China; xieyoulong1988@hotmail.com; 7 Department of Orthopaedics, Union Hospital, Tongji Medical College, Huazhong University of Science and Technology, Wuhan 430022, China; 18771036347@163.com; 8 The Faculty of Electrical Engineering and Computer Science, Ningbo University, Ningbo 315211, China; nano.plasma.uv@gmail.com; 9 Department of Orthopedic Surgery, The First Affiliated Hospital, Zhejiang University School of Medicine, Hangzhou 310003, China

**Keywords:** PNIPAM hydrogel, electrically conductive hydrogel, human motion detection, actuator, wound dressing

## Abstract

Poly(N-isopropylacrylamide) (PNIPAM) based electrically conductive hydrogels (PNIPAM-ECHs) have been extensively studied in recent decades due to their thermal-responsive (leading to the volume change of hydrogels) and electrically conductive performance. The incorporation of conductive components into the PNIPAM hydrogel network makes it become conductive hydrogel, and as a result, the PNIPAM hydrogel could become sensitive to an electrical signal, greatly expanding its application. In addition, conductive components usually bring new stimuli-responsive properties of PNIPAM-based hydrogels, such as near-infrared light and stress/strain responsive properties. PNIPAM-ECHs display a wide range of applications in human motion detection, actuators, controlled drug release, wound dressings, etc. To summarize recent research advances and achievements related to PNIPAM-ECHs, this manuscript first reviews the design and structure of representative PNIPAM-ECHs according to their conductive components. Then, the applications of PNIPAM-ECHs have been classified and discussed. Finally, the remaining problems related to PNIPAM-ECHs have been summarized and a future research direction is proposed which is to fabricate PNIPAM-ECHs with integrated multifunctionality.

## 1. Introduction

Schild et al. [1] reported poly(N-isopropylacrylamide) (PNIPAM) for the first time in 1956, and Scarpa et al. [2] subsequently discovered that PNIPAM displayed a temperature-induced phase transition phenomenon in 1967. Since then, PNIPAM has become a commonly used polymer for the preparation of temperature-sensitive smart materials [3,4,5]. Many scientific researchers have carried out research on PNIPAM because of its temperature-sensitive property, and thousands of related papers have been reported [6,7].

**Figure 1:**
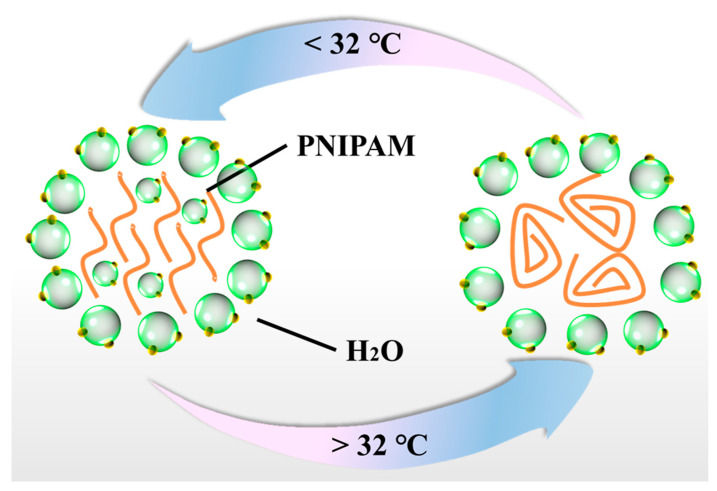
Scheme of phase transition behavior of PNIPAM.

The PNIPAM possesses a typical amide bond (-CO-NH-) and isopropyl group (-CH(CH3)_2_) structure: the amide bond is hydrophilic, and the isopropyl group is hydrophobic [8]. The PNIPAM aqueous solution can exhibit phase transition behavior around 32 °C as shown in Figure 1. The hydrogen bond interaction between the amide bond and water is strong and the hydrophobic interaction between the isopropyl group and water is weak when the temperature is lower than 32 °C, and as a result, the PNIPAM chain can be dissolved in water. When the temperature is higher than 32 °C, the hydrogen bond interaction between the amide bond and water is weakened and the hydrophobic interaction between the isopropyl group and water is enhanced, and the PNIPAM chain starts to be agglomerated, leading to phase separation [9]. The phase transition temperature of PNIPAM can be adjusted by chemical or physical modification. Generally speaking, the introduction of hydrophilic components can increase its phase transition temperature, and the introduction of hydrophobic components can reduce its phase transition temperature. PNIPAM-based hydrogels (PNIPAM-Hs) can exhibit a significant volume transition behavior (swelling/shrinking) around 32 °C due to the thermal responsive characteristic of PNIPAM [10,11,12]. At the same time, the PNIPAM-Hs also displayed changes of transparency during the swelling/shrinking process [13,14,15]. Due to the specific thermal responsive property of PNIPAM-Hs, great efforts have been made by researchers to investigate PNIPAM-Hs, and they have been extensively reported as drug/cell delivery vehicles [16,17,18,19,20], tissue engineering scaffolds [21,22,23], soft actuators [24,25,26,27], etc.

**Figure 2:**
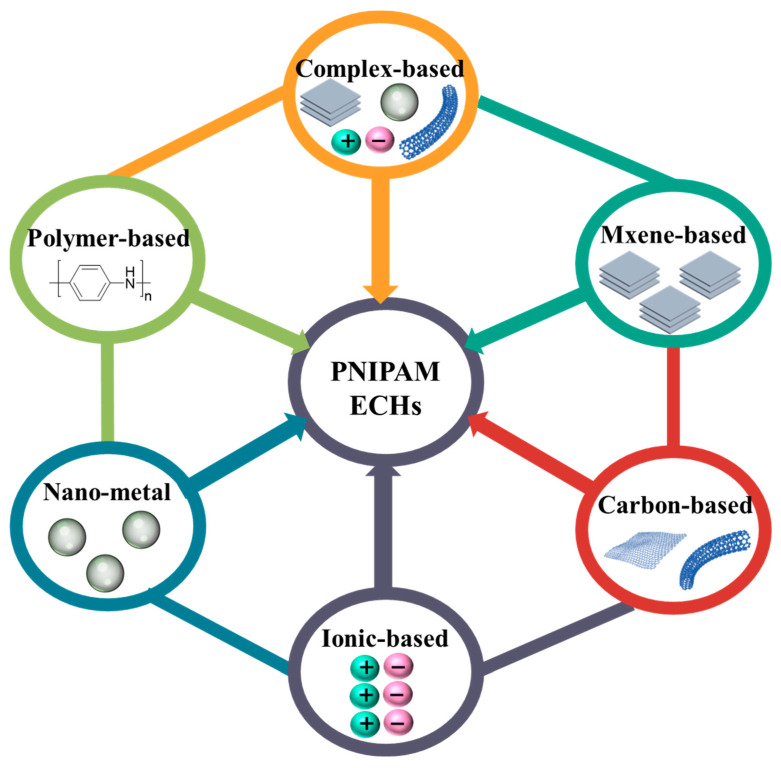
Scheme of 6 categories of PNIPAM-ECHs based on their conductive components.

Electrically conductive hydrogels possess both flexibility (originating from hydrogel) and electrical conductivity (originating from conductive components) [28,29,30,31]. Electrically conductive hydrogels can be divided into metal-based (gold nanoparticles and silver nanowires, etc.) conductive hydrogel [32,33,34,35,36], polymer-based conductive hydrogel (PANI, PPY, and PT, etc.) [37,38,39,40,41], carbon materials based (carbon nanotubes and graphene, etc.) conductive hydrogel [42,43,44,45], MXene-based conductive hydrogel [46,47,48], ionic conductive hydrogel (lithium chloride and sulfonate, etc.) [49,50,51,52] or multiple conductive substrates conductive hydrogel [53,54,55] according to their conductive components shown in Figure 2.

**Figure 3:**
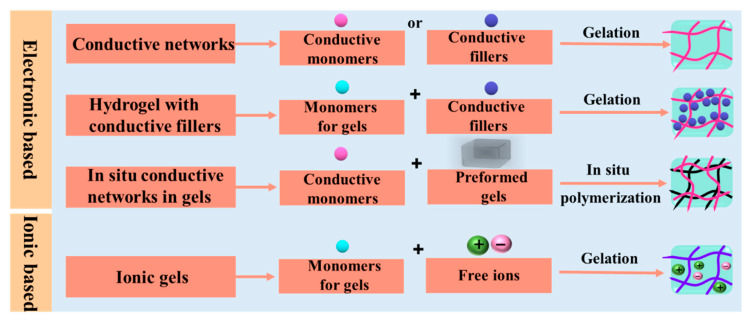
Scheme of preparation methods of conductive hydrogels. Copyright from Ref. [68] Royal Society of Chemistry.

The preparation method of electrically-conductive hydrogel is shown in Figure 3. The basic idea is to introduce conductive components into the hydrogel matrix [56,57,58,59]. The first preparation method is that conductive components form a hydrogel by themselves. For example, Bao et al. [60] used phytic acid as the crosslinking agent and monomer aniline as the raw material to crosslink polyaniline resulting in the formation of a conductive hydrogel. The second method is that the conductive component and hydrogel precursor are first mixed uniformly, and then the hydrogel precursor crosslinks to form a three-dimensional hydrogel network structure within conductive components [61,62,63,64]. For example, carbon nanotubes (CNTs) are modified and grafted with hydrophilic groups or functional groups to improve their dispersibility, and then hydrogel precursors are crosslinked with CNTs to construct conductive hydrogels. The third method is that conductive monomers in situ polymerize to form conductive polymers in a non-conductive hydrogel matrix to form a binary conductive hydrogel. For example, Yu et al. [65] prepared PNIPAM/PANI and PNIPAM/PPY binary conductive hydrogel, where PANI or PPY is formed after the PNIPAM hydrogel adsorbs the aniline or pyrrole monomers and then conductive monomers polymerized. The last method is to prepare ionic conductive hydrogels, where conductive ions can originate from the solvent or raw materials of the hydrogel [66,67]. The conductive hydrogels prepared by the first three methods usually show an opaque dark color (black or dark green), and the ionic conductive hydrogels prepared by the last method usually demonstrate a transparent light color. For different occasions, there will be different requirements for the transparency of the hydrogel, and researchers can choose a suitable preparation method to meet their application needs.

To combine the advantages of PNIPAM-based hydrogels and electrically conductive hydrogels, researchers have designed and prepared PNIPAM-based electrically conductive hydrogels (PNIPAM-ECHs). Conventional PNIPAM hydrogels don’t show electrical conductivity which could only be sensitive to temperature change, while the incorporation of conductive components into the PNIPAM hydrogel network makes it become conductive hydrogel, as a result, PNIPAM hydrogel could be sensitive to an electrical signal, greatly expanding the application scenarios of them. In addition, the majority of conductive components usually demonstrate photothermal properties (absorb NIR light and turn it into heat). The incorporation of conductive components into PNIPAM hydrogels could endow their NIR-light responsive property. On the other hand, there will be an electrical conductivity change of hydrogels when mechanical stimuli such as strain/stress are applied to PNIPAM-ECHs. Therefore, PNIPAM-ECHs can respond to mechanical stimuli. The introduction of conductive components usually endows new stimuli-responsive characteristics such as near-infrared (NIR) light and stress/strain stimuli-responsive properties [69,70,71], which are favored for specific applications of PNIPAM-ECHs. Currently, the applications of PNIPAM-ECHs focus on wearable electronic devices for human activity detection [72,73,74,75,76,77], soft robots and actuators [78,79,80,81,82], on-off switch [65,70,83,84,85,86], controlled drug release [87], photothermal therapy [88], and wound healing and closure [89,90,91], etc. To summarize the research progress related to PNIPAM-ECHs in the past decades, the preparation and performance of PNIPAM-ECHs are first classified and depicted according to their conductive components. Then, the applications of PNIPAM-ECHs in human motion detection, soft actuators, wound healing and closure, and drug release are discussed. Finally, the remaining problems and future research direction of PNIPAM-ECHs are concluded and directed.

## 2. Preparation and Performance of PNIPAM-ECHs

This section is mainly focused on the preparation and performance of PNIPAM-ECHs. Based on the conductive components of PNIPAM-ECHs shown in Table 1, they can be divided into a conductive polymer, carbon material, MXene material, metal nanoparticles, ions, and multiple conductive components based PNIPAM-ECHs. The design, preparation method, mechanical property, and electrical conductivity were summarized comprehensively.

### 2.1. PNIPAM-ECHs with Conductive Polymer Served as Conductive Components

**Figure 4:**
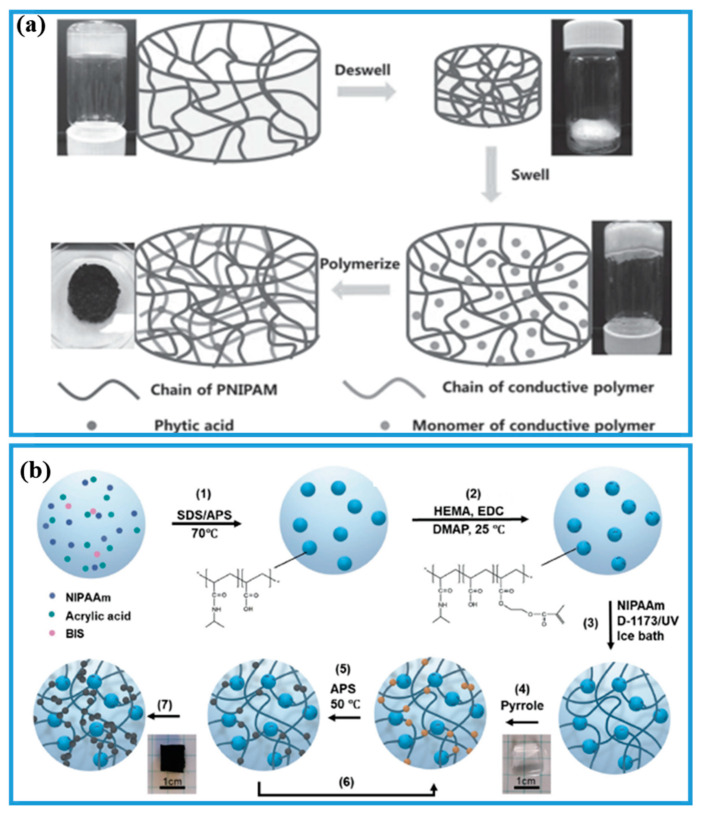
(**a**) Illustration of preparation of PNIPAM-PANI and PNIPAM-PPY double network PNIPAM-ECHs. Copyright from Ref. [65] John Wiley and Sons. (**b**) Illustration of preparation of well-distributed PPY PNIPAM-ECHs. Copyright from Ref. [100] Elsevier.

Since the discovery and development of conductive polymers, they have prompted ever-increasing interest in scientific research [92,93,94,95]. Conductive polymers usually exhibited brittle and rigid mechanical properties which might block their way in flexible sensors, energy storage, and tissue engineering scaffolds utilization [95,96]. To overcome those defects, significant advances by researchers have been made, and one of the ways is by introducing conductive polymers into a soft hydrogel matrix [97,98,99]. For example, Yu and coworkers [65] demonstrated double network PNIPAM-ECHs composed of PNIPAM-PANI and PNIPAM-PPY. As shown in Figure 4a, they first prepared PNIPAM hydrogel and then deswelled PNIPAM hydrogel was placed in conductive monomer aniline or pyrrole solution to absorb conductive monomer followed by polymerization of aniline or pyrrole monomer with phytic acid as crosslinker. The obtained PNIPAM-PANI and PNIPAM-PPY hydrogels displayed a compressive modulus of 66.1 and 54.5 Pa and conductivity of 0.2 S/m and 0.8 S/m, respectively. However, the mechanical tensile property of PNIPAM-PANI and PNIPAM-PPY hydrogels were not displayed. PNIPAM-PANI and PNIPAM-PPY hydrogels showed a slow deswelling/swelling speed, taking more than 10 h to complete a deswelling/swelling circle.

To solve the slow temperature-responsive speed of PNIPAM-ECHs, the cryogel method was used to fabricate PNIPAM-ECHs with rapid temperature-responsive speed. The cryogel was prepared under the melting point of the solvent, while solute polymeric precursors crosslinked and interconnected large macropores formed to obtain cryogel. Guo and coworkers [101] first prepared an interconnected macro-porous PNIPAM cryogel at −20 °C with rapid deswelling/swelling speed, followed by introducing PANI or PPY nanoparticles into the PNIPAM cryogel. The prepared PNIPAM-ECHs could accomplish their deswelling/swelling behavior within 2 min, demonstrating a breakthrough for the preparation of PNIPAM-ECHs with fast temperature-responsive speed. But the above method might cause poor distribution of conductive polymers (rich shell and poor core of conductive polymers) in the hydrogel matrix. Therefore, He and coworkers [100] designed a new type of PNIPAM-ECHs to fabricate well distribution of conductive polymers shown in Figure 4b. They first synthesized poly(NIPAM-co-AA) nanogel and then 2-hydroxyethyl methacrylate (HEMA) were reacted with poly(NIPAM-co-AA) to obtain C=C bond functionalized poly(NIPAM-co-AA) nanogel. The NIPAM was further polymerized and crosslinked by HEMA functionalized poly(NIPAM-co-AA). Finally, the PNIPAM hydrogel was immersed in pyrrole DMSO solution to absorb pyrrole because DMSO solvent could reduce the polymerization rate of pyrrole and simultaneously PNIPAM would not shrink when the temperature surpassed 32 °C, and as a consequence, the well distributed PPY of PNIPAM-ECH was carefully fabricated.

Generally speaking, conductive polymers are good candidates for fabricating PNIPAM-ECHs but the electrical conductivity and morphology of conductive polymers are not easy to be regulated, which should be further ameliorated.

### 2.2. PNIPAM-ECHs with Carbon Materials Served as Conductive Components

**Figure 5:**
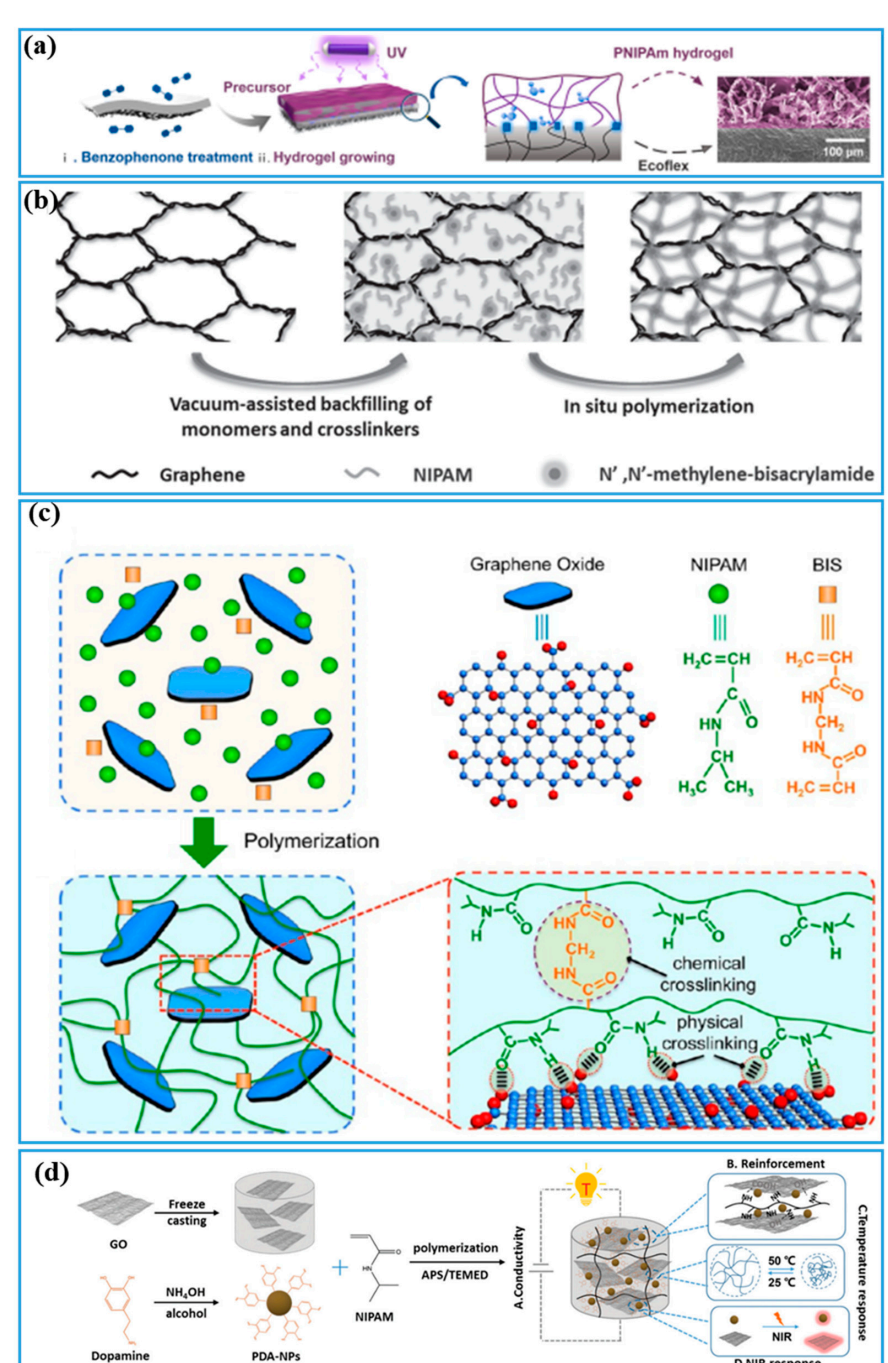
(**a**) Illustration of preparation process of PNIPAM/Ecoflex/CNTs hydrogel. Copyright from Ref. [72] Elsevier. (**b**) Illustration of preparation of PNIPAM and graphene aerogel double network hydrogel. Copyright from Ref. [105] John Wiley and Sons. (**c**) Illustration of preparation of PNIPAM with GO as conductive component reinforcement hydrogel. Copyright from Ref. [86] ACS publications. (**d**) Illustration of preparation of PNIPAM with rGO as conductive component and reinforcement hydrogel. Copyright from Ref. [90] Royal Society of Chemistry.

Carbon nanotubes (CNTs) and graphene oxide (GO) nanofillers are commonly used carbon materials for the preparation of ECHs. Among them, CNTs is one-dimensional nanotubes made of carbon that has a large aspect ratio and high electrical conductivity with high elastic modulus and stiffness [102,103]. The researchers consequently integrate CNTs into a soft flexible hydrogel network to construct conductive flexible hydrogel, including PNIPAM-ECHs. For instance, Hsiue and coworkers [104] fabricated PNIPAM-ECHs with multi-walled carbon nanotubes (MWCNTs) as conductive filler and polyethylene glycol dimethacrylate as crosslinker. However, the mechanical property and electrical conductivity were not comprehensively characterized. Apart from nanofillers, CNTs can also be used as a conductive substrate to construct PNIPAM-ECHs as demonstrated in Figure 5a [72]. They first prepared a CNTs film and Ecoflex/CNTs film, then afterward, PNIPAM hydrogel precursor was sprayed on Ecoflex/CNTs film and crosslinked. They obtained a composite hydrogel that demonstrated strong adhesiveness between film and hydrogel with a decent tensile strength of 12 kPa and tensile strain of 350%. But the composite hydrogel displayed high resistance of 1.5 MΩ, which is much higher than other PNIPAM-ECHs.

Because CNTs are hydrophobic and insoluble in water, the dispersion of CNTs should be improved for preparing hydrophilic conductive hydrogel. Chemical modification of hydrophilic groups (-OH and –COOH, etc.) on CNTs surface are frequently applied to improve the dispersity of CNTs and interfacial physical interactions (hydrogen bonding and π-π interaction) between CNTs and hydrogels.

Graphene is a two-dimensional carbon material consisting of a single layer of carbon atoms with hexagonal lattice nanostructure [106]. It is the thinnest carbon material with high electrical conductivity and tensile strength. Scientists also prepared PNIPAM-ECHs with graphene as a conductive component and mechanical reinforcement. As a typical example, researchers prepared mechanically robust PNIPAM-ECHs with a double network structure shown in Figure 5b [105]. They prepared graphene aerogel as the first network and conductive component at the same time. Then, the second PNIPAM hydrogel network was fabricated after graphene aerogel absorb the NIPAM solution. Similar to CNTs, graphene is hydrophobic and scientists prepared graphene oxide (GO) with many –OH, -COOH, and epoxy groups on GO nanosheets which could be soluble in water uniformly due to their strong physical interactions with water [107,108]. Lots of PNIPAM/GO hydrogel were reported by in situ polymerization methods in past decades. The obtained PNIPAM/GO hydrogel demonstrated an enhanced mechanical property compared to pure PNIPAM hydrogel displayed in Figure 5c [86]. However, GO sacrificed the electrical conductivity of graphene because the oxidation process greatly destroyed its conjugated structure, resulting in unsatisfied electrical conductivity. Therefore, partially reduced GO (rGO) was designed with better electrical conductivity compared to GO because of a reshaped conjugated structure without losing solubility and hydrophilicity. As a typical example shown in Figure 5d [90], the polydopamine (PDA) nanoparticles (NPs), GO and NIPAM were polymerized to prepare PNIPAM-ECHs, GO could be partially reduced by PDA nanoparticles resulting in rGO during the preparation process. The rGO could not only endow electrical conductivity but also provide mechanical reinforcement of PNIPAM hydrogel. It should be noticed that PDA would consume free radicals so more of an initiator, such as APS, should be added [109].

Although numerous advances of GO and rGO-based PNIPAM-ECHs have been achieved, some of the drawbacks couldn’t be ignored. First, the electrical conductivity of GO and rGO couldn’t compete with CNTs, graphene, and other conductive fillers. What is more, the oxidation and reduction reaction of graphene is complicated and time-consuming. Preparation of GO with high electrical conductivity and efficacy is meaningful and challenging.

### 2.3. PNIPAM-ECHs with MXene Served as Conductive Components

**Figure 6:**
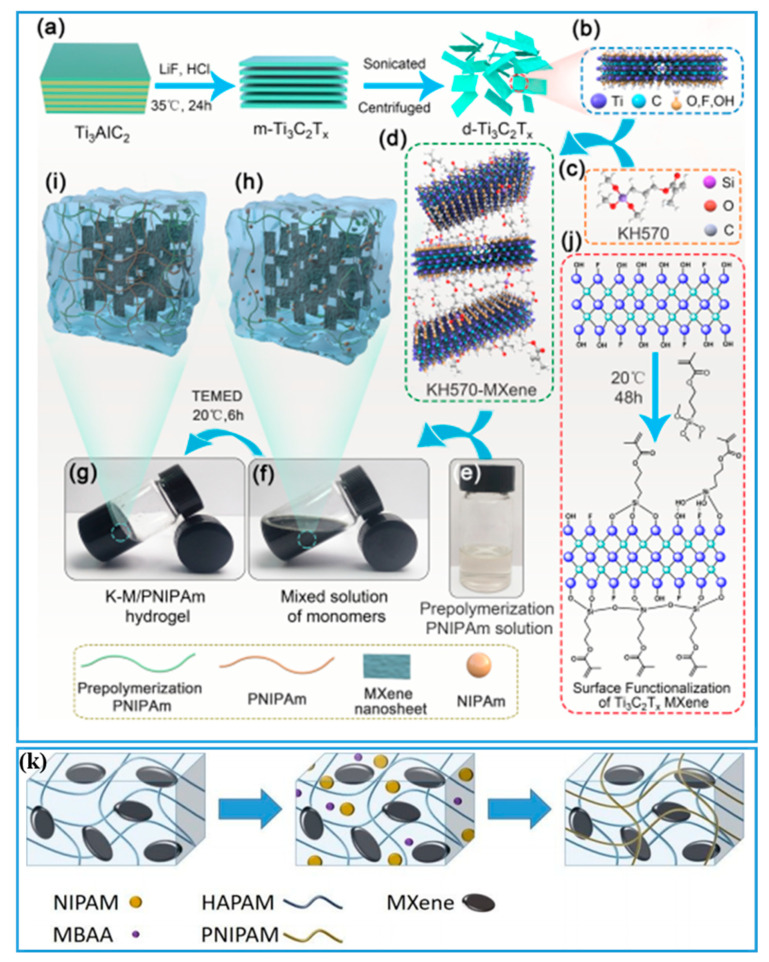
(**a**) Illustration of preparation process of Ti_3_C_2_T_x_ Mxene. (**b**) Structure of Ti_3_C_2_T_x_ Mxene. (**c**) Structure of KH570. (**d**) Structure of KH570-Mxene. (**e**) Picture of PNIPAM-PAM solution. (**f**) Picture of mixed solution with MXene. (**g**) Picture of prepared PNIPAM-ECH. (**h**) Structure of mixed solution with MXene. (**i**) Structure of prepared PNIPAM-ECH. (**j**) Illustration of surface functionalization of MXene. Copyright from Ref. [121] ACS publications. (**k**) Illustration of preparation of double network PNIPAM-ECH. Copyright from Ref. [120] ACS publications.

MXene is a two-dimensional material composed of transition metal carbide and/or nitride was first reported in 2011 [110]. There are lots of hydrophilic groups such as –OH groups on the MXene surface so that they can be soluble in water easily [111,112,113]. Besides, MXene also demonstrates metal-like electrical conductivity without disposing of its hydrophilicity [114,115]. It has been extensively used for building PNIPAM-ECHs in the past due to MXene’s fantastic characteristics [116,117]. First, MXene can have strong physical interactions with the hydrogel network thus it can provide enhanced mechanical properties of the hydrogel. Besides, the distribution of MXene in the hydrogel network is uniform and even, without aggregation, so that the conductive pathway is stable and sensitive. The general preparation process of the MXene nanosheet is described as follows [118,119]: a typical “MAX” (M represents the transition metal, A refers to IIIA and IVA elements, X is C and/or N) was chemically etched by solution containing F^-^, and consequently, MXene was built. During etching, the A in “MAX” was selectively removed resulting in the MXene nanosheet. As a typical example shown in Figure 6k, Ran and coworkers [120] first prepared supramolecular hydrogel using acrylamide (AM), OP-10 emulsifier, and lauryl methacrylate in Ti_3_C_2_ MXene water solution followed by lyophilization. Then, the lyophilized hydrogel was put in PNIPAM hydrogel precursor solution to take it in completely, followed by slow polymerization of PNIPAM at low temperature for 7 days to obtain a binary network of PNIPAM-ECHs. The double network PNIPAM-ECH displayed a stretchability of 1400%, a tensile strength of 0.4 MPa, and electrical conductivity of ~1.1 S/m with self-healing property. To further ameliorate the compatibility between MXene and PNIPAM, MXene could be surfaced modified by a silane coupler displayed in Figure 6a–j [121]. Researchers first prepared Ti_3_C_2_T_x_ MXene by HF acid etching followed by sonication and centrifugation, then MXene was functionalized with γ-methacryloxypropyltrimethoxysilane (KH570) to improve compatibility with PNIPAM. Second, NIPAM and AM were copolymerized to form a PNIPAM-PAM solution. Third, surface modified MXene solution was mixed with PNIPAM-PAM solution followed by adding NIPAM, initiator, and crosslinker to fabricate PNIPAM-ECHs. The prepared PNIPAM-ECHs displayed a desirable strain sensitivity with a gauge factor of ~4.5.

Some limitations of MXene are unavoidable in the ever-increasing development of PNIPAM-ECHs. First of all, the preparation of MXene is complicated. Moreover, dangerous and harmful chemicals such as HF acid might be applied for etching. We should be very careful when we use HF acid because HF acid could not only harm humans but also the environment. Therefore, it is demanded and significant to come up with new ways to generate MXene safely and simply.

### 2.4. PNIPAM-ECHs with Metal Nanoparticles Served as Conductive Components

**Figure 7:**
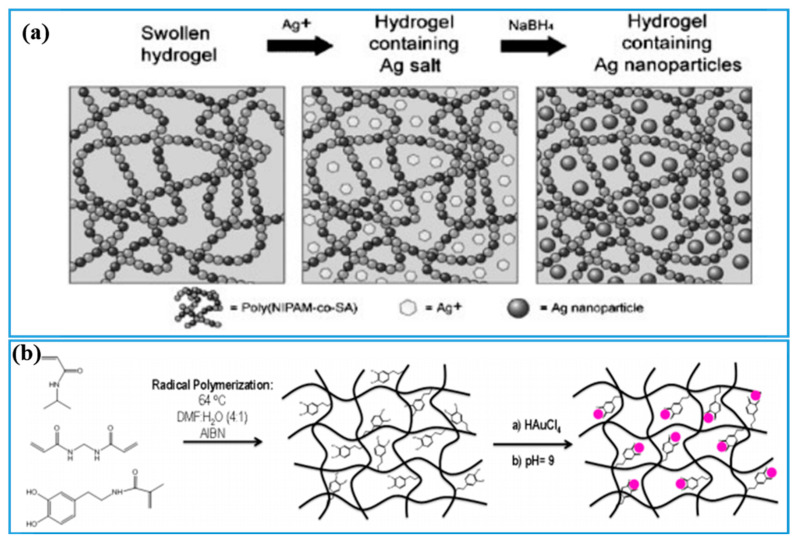
(**a**) Illustration of preparation process of PNIPAM-ECH with Ag nanoparticles served as conductive filler. Copyright from Ref. [125] John Wiley and Sons. (**b**) Illustration of preparation process of PNIPAM-ECH with Au nanorods served as conductive filler. Copyright from Ref. [126] ACS publications.

Metal is a highly conductive material and it is usually designed in nanoparticle (Ag or Au nanoparticle), nanosliver (Ag nanosliver), and nanorod (Au nanorod) forms to fabricate PNIPAM-ECHs [122,123]. The metal nanoparticles could be prepared by adding extracts to metal salt solutions and metal ions are reduced to metal nanoparticles [124]. Au and Ag nanomaterials are frequently used to fabricate PNIPAM-ECHs because they possess extra biological functions such as antibacterial and photodynamic therapy properties. As a proof of concept shown in Figure 7a [125], NIPAM and sodium acrylate (SA) were first copolymerized to obtain poly(NIPAM-co-SA) hydrogel, and then poly(NIPAM-co-SA) hydrogel was placed in Ag salt solution to swell Ag salt completely, followed by adding NaBH_4_ to reduce Ag^+^ to Ag nanoparticles. Au nanorods were incorporated in a similar way shown in Figure 7b, where Marcelo and coworkers [126] first copolymerized catechol-methacrylamide and NIPAM to obtain hydrogel and then hydrogel swelled the HAuCl_4_ solution quickly and Au^+^ reduced to Au gradually by catechol groups to obtain PNIPAM-ECH. Although nano metals demonstrated high electrical conductivity, their distribution in hydrogel still needs to be regulated, and precious metals such as Au and Ag are expensive which would increase the cost of PNIPAM-ECHs.

### 2.5. PNIPAM-ECHs with Ion Served as Conductive Components

**Figure 8:**
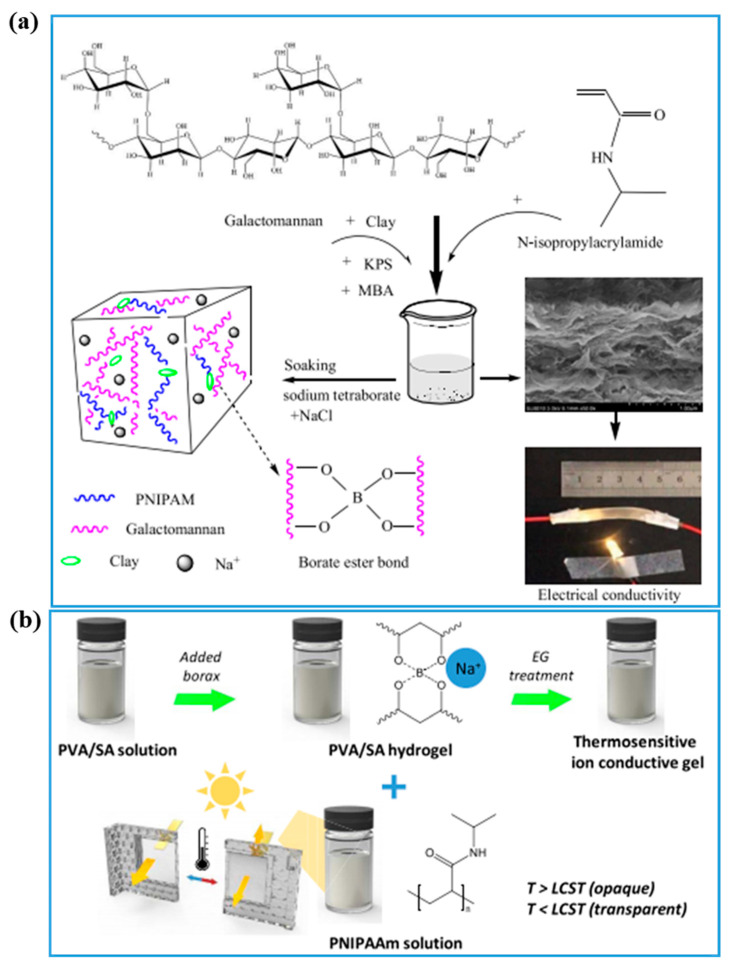
(**a**) Illustration of preparation process of PNIPAM-ECH with Na^+^ served as conductive filler. Copyright from Ref. [128] ACS publications. (**b**) Illustration preparation process of PNIPAM-ECH with clay and Na^+^ served as conductive filler. Copyright from Ref. [77] ACS publications.

The above listed conductive components are electronically conductive and prepared ECHs which usually displayed a dark color. Apart from the electronic conductive mechanism, the ions [127] could also be used for the preparation of PNIPAM-ECHs with a light and transparent appearance. This kind of morphology and color are suitable for some utilizations such as wound dressing; thus the wound healing process is visible. The ions can come from the polyelectrolytes and/or salts solution. As shown in Figure 8a, Duan and coworkers [128] first fabricated a hydrogel by copolymerization of NIPAM and galactomannan with clay as mechanical reinforcement. Then, the hydrogel was placed in NaCl and sodium borate solution to obtain binary network ionic conductive PNIPAM-ECH. The obtained PNIPAM-ECHs displayed enhanced mechanical performance after being treated with NaCl and sodium borate solution with the highest modulus of ~60 MPa, elongation at break of 1370%, and electrical conductivity of 4.40 S/m. Free ions from salt solution might show poor retention in the hydrogel matrix resulting in a decrease in electrical conductivity. Therefore, ions from polymer are also involved in the preparation of PNIPAM-ECHs demonstrated in Figure 8b [77]. The sodium polyacrylate solution was mixed with PVA solution gradually, then PNIPAM was added to the above mixture solution followed by adding borax to crosslink and form PNIPAM-ECHs. The prepared PNIPAM-ECHs exhibited a modulus of 423 kPa, elongation at break of ~600%, and electrical conductivity of 6.64 × 10^−2^ S/m, which is much lower than clay reinforced hydrogel. What’s more, nanoclay of laponite could also be used to fabricate ionic PNIPAM-ECHs because it can release Na^+^. In addition to provide ionic electrical conductivity, they can be served as nano-reinforcement to enhance the mechanical property of hydrogels significantly whereby strong physical interactions (hydrogen bonding and electrostatic interaction) between hydrogel and laponite formed.

### 2.6. PNIPAM-ECHs with Multiple Components as Conductive Components

**Figure 9:**
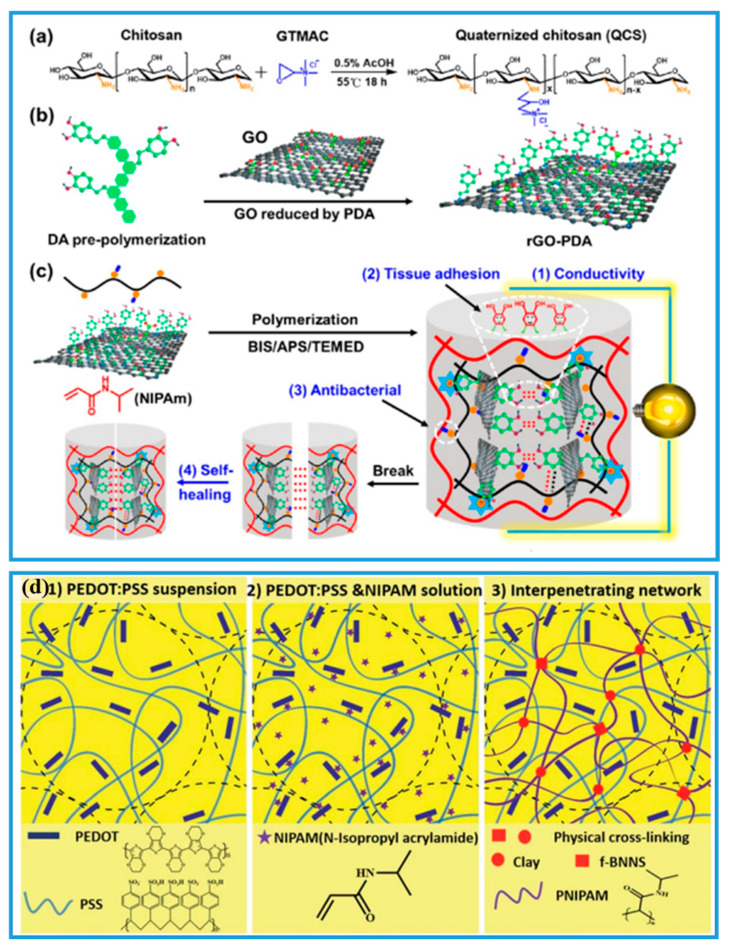
(**a**) Illustration of preparation process of quaternized chitosan. (**b**) Illustration of preparation process of rGO-PDA. (**c**) Illustration of preparation process of PNIPAM-ECH with quaternized chitosan and rGO-PDA served as conductive components. Copyright from Ref. [89] ACS publications. (**d**) Illustration of preparation process with PEDOT:PSS and clay served as conductive components. Copyright from Ref. [73] Royal Society of Chemistry.

In addition to the single conductive component of PNIPAM-ECHs, the multiple conductive components were also designed by researchers because they might not only improve the electrical conductivity but also enhance the mechanical performance as well as bring new stimuli-responsive characteristics and functions of PNIPAM-ECHs [43,76,129,130]. As a typical example shown in Figure 9a–c, Guo and coworkers [89] prepared double networks of PNIPAM-ECHs using quaternized chitosan (QCS), rGO-PDA, and NIPAM. The PNIPAM could form the first network by BIS crosslinker, and the second network was built between rGO-PDA and quaternized chitosan through a Schiff-base reaction. The second network contributed to the antibacterial (inherent and photothermal) and self-healing properties of the hydrogel as well as tissue adhesiveness, which significantly broaden the application of PNIPAM-ECHs in the biomedical field. Apart from introducing a new function, the mechanical property of PNIPAM-ECHs could also be enhanced, for instance, as proof shown in Figure 9d, Xu and coworkers [73] prepared strong physical crosslinked PNIPAM-ECHs with self-healing, adhesive, and photothermal responsive properties by using NIPAM, PEDOT:PSS, functionalized boron nitride nanosheets (f-BNNS), and nanoclay. The constructed hydrogel displayed a maximum strain of ~2600% and it can even withstand 90% compressive strain without mechanical failure, demonstrating superior mechanical performances. These results were 2 times higher than their previously reported PNIPAM/f-BNNS/nanoclay hydrogel. Because PSS could form strong hydrogen bond interactions with PNIPAM and f-BNNS, and other physical interactions such as electrostatic interactions, π-π static interactions existed between PEDOT and PSS. Moreover, the hydrogel displayed enhanced electrical conductivity compared to pure PEDOT:PSS.

The six categories of PNIPAM-ECHs based on their conductive components are comprehensively summarized. The incorporation of conductive components made a huge difference in PNIPAM hydrogel function. First, the introduction of electrically conductivity ensures PNIPAM-ECHs could be applied in the electronic field such as conductive sensors and on-off switches. Second, the electronic conductive components usually exhibited photothermal behavior so that PNIPAM-ECHs have NIR-light responsive properties at the same time. PNIPAM-ECHs could be served as NIR-light-controlled actuators or soft robots after introducing NIR-light responsive conductive components. Third, conductive components can also be used as mechanical reinforcement as they greatly improve the mechanical performance of PNIPAM-ECHs. Other properties such as self-healing, adhesive and shape memory properties can also be achieved by PNIPAM-ECHs to develop integrated multifunctional PNIPAM-ECHs, greatly expanding their application scenarios.

**Table 1:** Representative PNIPAM-ECHs based on their conductive components. “-” means not determined.

Gel Code	Conductive Components	Electrical Conductivity or Resistance	Applications	Reference
1	PANI or PPY	0.8 S/m	On-off switch	[65]
2	PANI or PPY	-	On-off switch, and pressure sensing	[101]
3	PANI	0.64 S/m	Repaired electric circuit, on-off switch	[70]
4	PANI	0.06 mS/cm	Actuator	[79]
5	PPY	-	Actuator	[78]
6	PPY	-	Actuator	[100]
7	MWCNTs	-	Scaffold	[104]
8	MWCNTs	1.5 MΩ	Sensor and actuator	[72]
9	MWCNTs	-	Photothermal therapy	[88]
10	Graphene	10 S/m	-	[106]
11	GO	-	On-off switch	[86]
12	rGO	-	Wound dressing	[90]
13	MXene	~1.1 S/m	Pressure sensing	[120]
14	MXene	~5.5 mS/cm	Human motion detection	[121]
15	MXene	-	Auto valve	[116]
16	Mxene		Actuator	[117]
17	Ag nanoparticles	-	-	[125]
18	Au nanoparticles	-	-	[126]
19	Au nanorods	-	On-off switch	[122]
20	Au nanorods	-	Drug delivery	[123]
21	Ions	4.40 S/m	Human motion detection	[128]
22	Ions	0.064 S/m,	Touch sensors	[77]
23	Ions	~0.5 S/m	Strain and pressure sensing	[127]
24	Ions	-	Human motion detection	[69]
25	Ions	-	Wireless human motion detection	[74]
26	MWCNTs and ions	0.19 S/m	Human motion detection and 3D printing	[43]
27	MWCNTs and PPY	35 S/m	Human motion detection and pressure sensing	[130]
28	Ions and rGO	5.6 mS/cm	Wound dressing	[89]
29	PEDOT:PSS and GO	0.08 S/m	Human motion detection	[75]
30	PEDOT:PSS and ions	-	Human motion detection	[73]

## 3. Applications of PNIPAM-ECHs

PNIPAM-ECHs were extensively applied in a variety of fields due to their advantages, including human motion sensors, soft actuators and bionic robots, on-off switch, photo-thermal therapy, drug delivery vehicles, and wound dressing. This section is mainly highlighted the biomedical application of PNIPAM-ECHs.

### 3.1. Human Motion Detection Application of PNIPAM-ECHs

**Figure 10:**
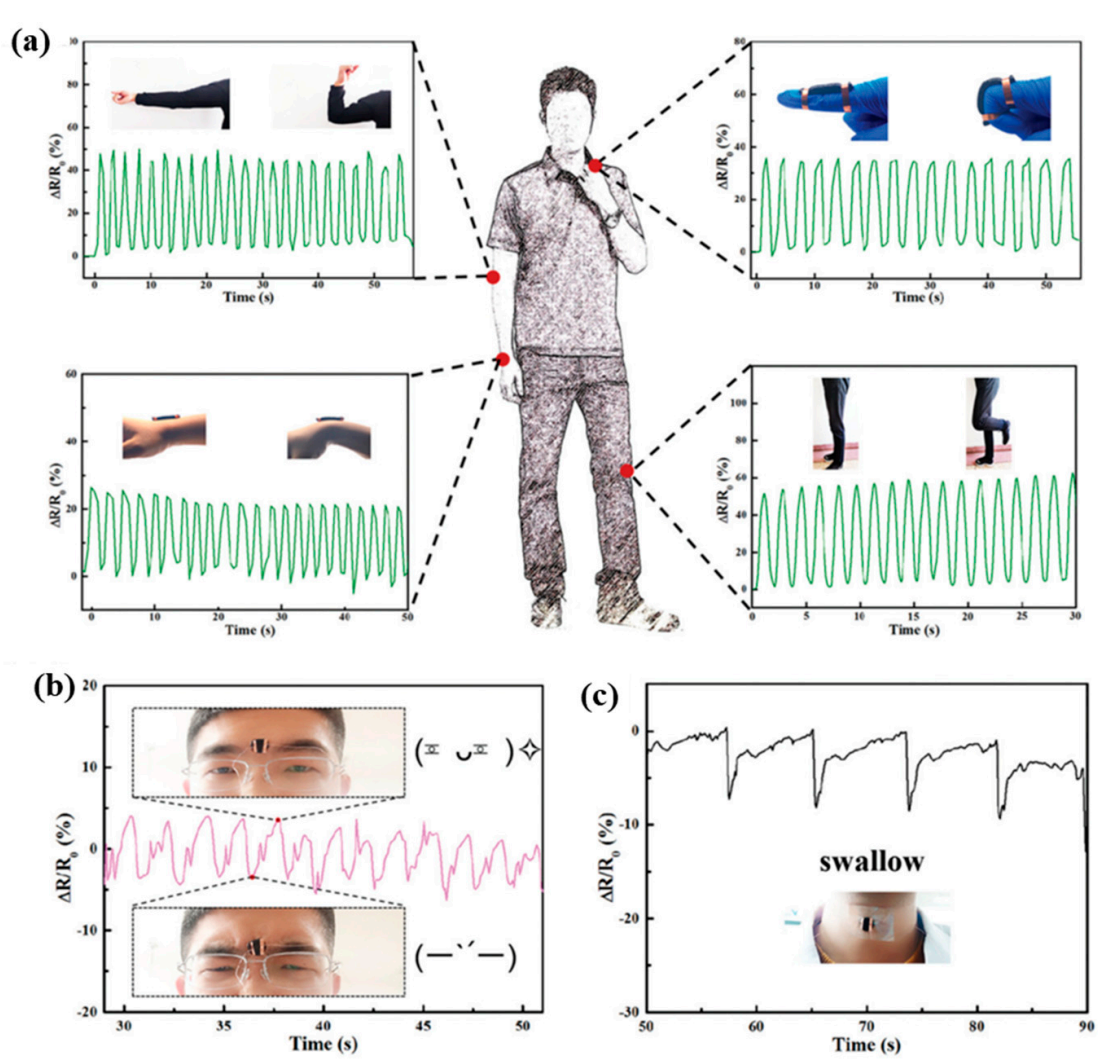
(**a**) PNIPAM-ECH for large-scale human motion detection (elbow motion, finger bending, wrist bending, and knee motion). (**b**) PNIPAM-ECH for facial expression detection. (**c**) PNIPAM-ECH for swallow motion detection. Copyright from Ref. [75] Royal Society of Chemistry.

Similar to other conductive hydrogel sensors, the PNIPAM-ECHs could also be used for human motion sensing and detection because of their potential excellent mechanical property. On the other hand, the body temperature would increase after human motion, resulting in an electrical conductivity change in the PNIPAM-ECHs. The hydrogel sensor fabrication and sensing mechanism is described as follows: the hydrogel is mounted on the human body to be tested and then the wire is connected to the hydrogel and detector. The hydrogel resistance would change as long as the tested part is moving because the strain and stress of hydrogel would change simultaneously. As a typical example in Figure 10a,b [75], large scale motion (including finger bending, wrist bending, elbow and knee motion, etc.) and small scale motion (swallow, facial expression and subtle motion, etc.) can both be detected. They prepared a PNIPAM-ECH using NIPAM and conductive components PEDOT:PSS and rGO. The obtained hydrogel exhibited a high tensile strain (~2500%) and strength (29 kPa) as well as adhesive property. They first characterized the large-scale motion including elbow, finger bending, wrist, and knee, which displayed resistance changes of ~50%, ~28%, ~38%, and ~50%, respectively. The facial expression and swallow motion were subsequently tested, which exhibited much smaller resistance changes compared to large-scale motion.

**Figure 11:**
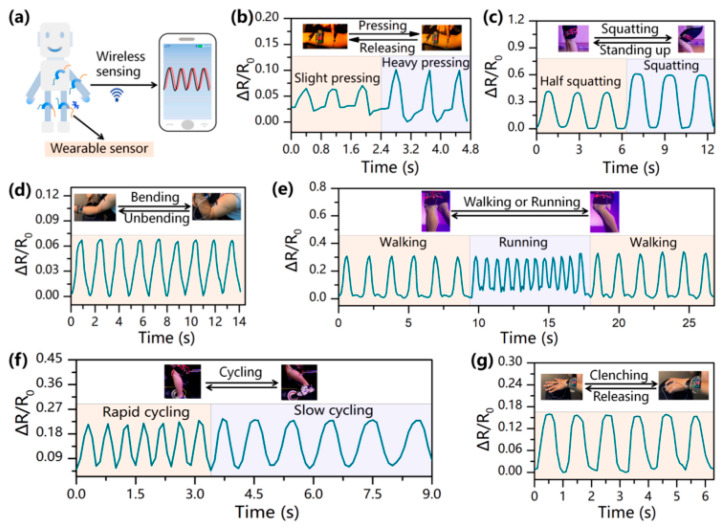
(**a**) Illustration of wearable sensor for wireless sensing. PNIPAM-ECH for (**b**) pressing-releasing detection. (**c**) squatting-standing up detection. (**d**) finger bending-unbending detection. (**e**) walking or running detection. (**f**) rapid cycling and slow cycling detection. (**g**) clenching and releasing detection. Copyright from Ref. [74] Elsevier.

It is inconvenient for human motion detection sometimes because the wire is mounted on the human body. Therefore, researchers developed wireless sensors by using PNIPAM-ECH shown in Figure 11 [74]. The PNIPAM-ECH consists of PVA, Poly(NIPAM-co-AM), Fe_3_O_4,_ and KCl, among them, PVA was introduced to improve the adhesiveness of hydrogel, Fe_3_O_4_ was the magnetic responsive component and KCl served as a conductive component. A Bluetooth transmission system was implanted in hydrogel so that it can transfer resistance change wirelessly. The pressing, squatting, bending, walking, or running, cycling, and clenching motions were characterized carefully. All of the motions demonstrated stable and regular resistance change.

Although there are lots of PNIPAM-ECHs applied in human motion detection, PNIPAM-ECHs applied in physiological signal monitoring (electrocardiogram (ECG) and electroencephalogram (EEG) signal) works have not been reported to the best of our knowledge. Human physiological signal monitoring is vital for human health so it is meaningful to design and prepare PNIPAM-ECHs for ECG and EEG signal detection. What is more, the lack of long-term stability and environmental tolerance would block their way on human motion detection, because PNIPAM-ECHs would lose water in a dry environment and freeze when the temperature is low. Environment tolerant PNIPAM-ECHs are required to be fabricated for human motion detection in special environments.

Currently, most of the PNIPAM-ECHs for sensors need an external energy supply such as batteries. Self-powered sensors based on PNIPAM-ECHs that maintain their sensing functions are designed and fabricated by the Park and In group [131]. They designed a solar cell system combined with a stress and strain sensor so that the self-powered system could detect human motion directly under sunlight.

### 3.2. Soft Actuators and Robotics Application of PNIPAM-ECHs

**Figure 12:**
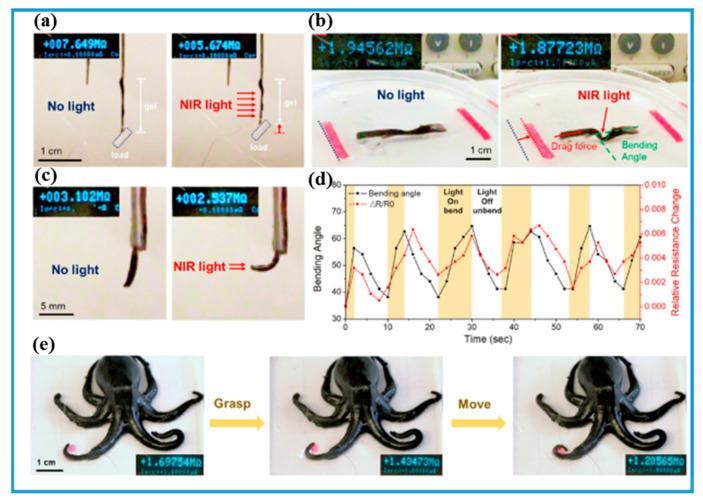
(**a**) Pictures of loading behavior of PNIPAM-ECH hydrogel triggered by NIR light. (**b**) Pictures of bending behavior of PNIPAM-ECH hydrogel triggered by NIR light. (**c**) Actuation behavior of octopus-like PNIPAM-ECH hydrogel. (**d**) Bending angle and resistance change curves of PNIPAM-ECH. (**e**) Actuation behavior of octopus-like PNIPAM-ECH hydrogel. Copyright from Ref. [100] Elsevier.

The actuator could convert stimuli signals or energy to motion. It requires an actuation force to allow for its movement and interaction with the external environment [132,133,134]. Soft actuator [135,136] is composed of flexible materials rather than rigid materials (metals, hard plastics, and ceramics), which is beneficial to compatibility with humans. Soft actuators can be applied as artificial muscles, medical devices, and soft grippers. PNIPAM-ECHs can convert heat stimuli to motion and electrical conductivity change at the same time, which is beneficial for real-time data recording for actuators and robotics applications. He and coworkers [79,100] recently designed and prepared two types of PNIPAM-ECHs for soft actuators and robotics. As shown in Figure 12a, actuation behavior induced by the NIR light of PNIPAM-ECH was first demonstrated. The PNIPAM-ECH would shrink after NIR light exposure and pull up the load at the same time. The bending behavior of PNIPAM-ECH was shown in Figure 12b where PNIPAM-ECH showed a maximum bending angle of ~65° when exposed to NIR light. The bending angle would turn to 40° after the light was switched off. What is more, the relative resistance change curve of PNIPAM-ECH corresponded to the bending angle curve displayed in Figure 12d, exhibiting stable and controllable actuation behavior induced by NIR-light. PNIPAM-ECH was further designed and fabricated into octopus-like hydrogel for soft robotics shown in Figure 12e. The octopus could grasp a pink ball after hydrogel exposure to NIR light. PNIPAM-ECH is a good candidate for soft actuator because of the thermal responsive property of PNIPAM. Moreover, the conductive components are usually NIR light-responsive so that PNIPAM-ECH could be manipulated by NIR light. However, the accuracy, responsive speed, and stability of PNIPAM-ECH soft actuators are not good enough to compare with conventional actuators due to the disadvantages of PNIPAM-ECHs. It is still a great challenge to fabricate PNIPAM-ECH soft actuators and robotics with rapid response speed and actuating stability.

### 3.3. Wound Dressing and Wound Closure Application of PNIPAM-ECHs

**Figure 13:**
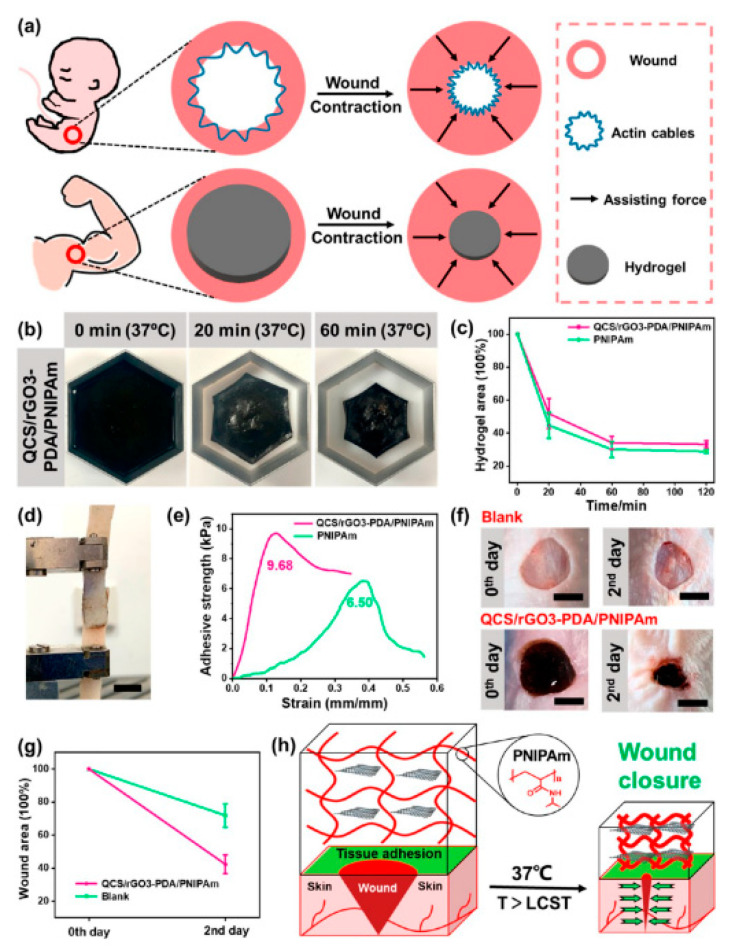
(**a**) Illustration of wound closure process of PNIPAM-ECH wound dressing. (**b**) Pictures of shrunk behavior of PNIPAM-ECH at 37 °C. (**c**) Change of hydrogel area of PNIPAM-ECH at 37 °C. (**d**) Picture of PNIPAM-ECH for adhesive strength testing. (**e**) Adhesive strength of PNIPAM-ECH. (**f**) Wound closure pictures of PNIPAM-ECH hydrogel group and blank group. (**g**) Wound area curves of PNIPAM-ECH hydrogel group and blank group. (**h**) Illustration of wound closure process of wound closure. Copy right from Ref. [89] ACS publications.

Wound dressings are used to clean and protect the wound from the external environment to promote wound healing [137,138,139]. The main purpose of wound closure is to stop wound bleeding, speed wound healing, and prevent wound infection [140,141,142]. Hydrogel dressing could keep moisture and absorb exudate to accelerate wound healing which was developed rapidly in recent years [143,144]. The PNIPAM-ECHs are also designed as wound dressings to promote wound healing and wound closure because PNIPAM-ECHs would shrink with body temperature which can induce wound contraction. In addition, electrical conductivity enables cellular signaling and function in many types of tissues such as human skin, cardiac tissue, muscle tissue, and nerve tissue. It plays a significant role in tissue function. On the other hand, some of the conductive components such as polyaniline possess good free radical scavenging capacity to reduce wound inflammation and enhance wound healing. As a typical example shown in Figure 13a by Guo and coworkers [89], the illustration of PNIPAM-ECH is used for wound dressing to promote wound healing and wound closure. Figure 13b,c demonstrated the shrinking behavior of PNIPAM-ECH at 37 °C. The adhesive property of PNIPAM-ECH is shown in Figure 13d,e. Wound closure results are shown in Figure 13f–h, the PNIPAM-ECH group displayed an obvious closure of the wound area compared to the blank group to promote wound healing. Other functions such as antibacterial and adhesive properties are usually integrated into PNIPAM-ECH to accelerate wound healing. However, the biosafety of PNIPAM-ECH dressing might be a problem, because PNIPAM and conductive components are not biodegradable, and NIPAM is toxic to humans. The biocompatibility of PNIPAM-ECH should be further evaluated before clinical utilization.

### 3.4. Drug Delivery Application of PNIPAM-ECHs

**Figure 14:**
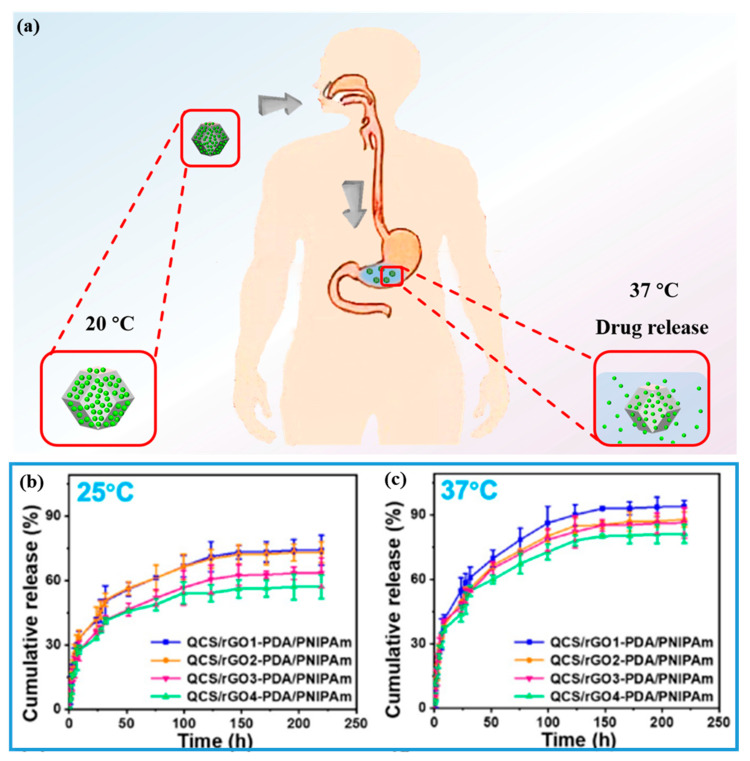
(**a**) Scheme of drug release process of PNIPAM-ECH in vivo. (b) Cumulative release of drug at (**b**) 25 °C and (**c**) 37 °C of PNIPAM-ECH. Copy right from Ref. [89] ACS publications.

Hydrogel drug delivery means that hydrogel could improve its biosafety and efficacy by controlling release rate, quantity, and place of drugs. The hydrogel drug delivery system should be stable, and delivery could be controlled under various physiological changes, as a result, increasing the bioavailability of the drug. PNIPAM-ECHs could be used for temperature and electrical-stimuli-dependent drug delivery vehicles due to their thermal and electrical responsive property. Figure 14a demonstrates the drug release process of PNIPAM-ECHs in vivo. Figure 14b,c [89] shows the tetracycline of doxycycline drug release curve at 25 °C and 37 °C, respectively. 57~74% (from QCS/rGO4-PDA/PNIPAM to QCS/rGO1-PDA/PNIPAM) of drug released after 10 days at 25 °C, while 81~93% (from QCS/rGO4-PDA/PNIPAM to QCS/rGO1-PDA/PNIPAM) of drug released after 10 days at 37 °C, exhibited higher drug release amount at 37 °C. This is because PNIPAM-ECHs would shrink at 37 °C and more drugs could release from the hydrogel matrix.

Although PNIPAM-ECHs could be used as drug delivery vehicles, PNIPAM-ECHs drug delivery vehicles also have some disadvantages. First, the release process of the drug is temperature-dependent, but the on-demand release of the drug is still needs to be regulated. Second, the biodegradability of PNIPAM is a problem for drug release studies in vivo, which could cause immune reactions in creatures. Third, how to maintain drug levels in a suitable therapeutic range should be further investigated.

## 4. Conclusions and Outlooks

In this review, the recent progress related to PNIPAM-ECHs is comprehensively summarized. The preparation and properties are first demonstrated and followed by the applications of PNIPAM-ECHs. Great strides in the use of PNIPAM-ECHs have been made, and PNIPAM-ECHs were successfully used in various settings, especially in human motion sensing, wound dressing, and soft actuators, and controlled drug release. However, there are still some problems that need to be resolved according to our recent study related to PNIPAM-ECHs: (1) The research of PNIPAM-ECHs for human physiological signal monitoring (such as ECG signals and EEG signals) is not yet sufficient. Such applications require hydrogels to possess high sensitivity to external stimuli signals and how to recognize those stimuli signals and respond to them accurately still needs to be explored; (2) PNIPAM-ECHs have drawbacks in their long-term stability and environmental tolerance. These defects may limit the application of PNIPAM-ECHs in extreme environments. (3) The accurate actuation of PNIPAM-ECHs still needs to be improved for hydrogel machine application. (4) Some of the raw materials of PNIPAM-ECHs are toxic, PNIPAM and conductive components are usually not biodegradable which may cause harm to the organism. Given these issues, the following prospects for future research are proposed:

(1) Preparation of PNIPAM-ECHs with high sensitivity and stability as flexible wearable sensing devices so that they can be better used for human ECG signal, EEG signal, and arterial pressure monitoring.
(2) Because the hydrogel is prone to dehydration/freezing in a dry/frozen environment, moisturizing and anti-freezing PNIPAM-ECHs are required to improve the long-term stability and ability to withstand the extreme environment of PNIPAM-ECHs.
(3) Accuracy and response speed of PNIPAM-ECHs need to be regulated for soft robots and actuators.
(4) For biomedical medical applications, the biocompatibility of PNIPAM-ECHs needs to be further evaluated.

## Data Availability

Not applicable.

## Abbreviations

PNIPAM	Poly(N-isopropylacrylamide)
PNIPAM-ECHs	Poly(N-isopropylacrylamide) based electrically conductive hydrogels
ECHs	electrically conductive hydrogels
PNIPAM-Hs	Poly(N-isopropylacrylamide) based hydrogels
PANI	Polyaniline
PPY	Polypyrrole
PT	Polythiophene
CNTs	Carbon nanotubes
NIR	Near-infrared
HEMA	2-Hydroxyethyl methacrylate
MWCNTs	Multi-walled carbon nanotubes
AA	Acrylic acid
DMSO	Dimethyl sulfoxide
GO	Graphene oxide
rGO	reduced Graphene oxide
SA	Sodium acrylate
PDA	Polydopamine
NPs	Nanoparticles
KH570	γ-methacryloxypropyltrimethoxysilane
f-BNNS	functionalized boron nitride nanosheets
PVA	Polyvinyl alcohol
PEDOT:PSS	Poly(3:4-ethylenedioxythiophene):polystyrene sulfonate
BIS N: N’	methylenebis(acrylamide)
QCS	Quaternized chitosan
APS	Ammonium persulphate
TEMED	Tetramethylethylenediamine
PAM	Polyacrylamide
AM	Acrylamide
ECG	Electrocardiogram
EEG	Electroencephalogram
